# Revisiting Javal’s rule: a fresh and improved power vector approach according to age

**DOI:** 10.1007/s00417-023-06195-x

**Published:** 2023-08-08

**Authors:** Raquel Salvador-Roger, César Albarrán-Diego, Nuria Garzón, María García-Montero, Gonzalo Muñoz, Vicente Micó, José J. Esteve-Taboada

**Affiliations:** 1 Department of Optics and Optometry and Vision Sciences, University of Valencia, 46100 Burjassot, Valencia Spain; 2 Department of Optometry and Vision, Universidad Complutense de Madrid, 28037 Madrid, Spain; 3 Marqués de Sotelo Ophthalmological Clinic, 46002 Valencia,, Spain; 4 Baviera Castellón Ophthalmological Clinic, 12003 Castelló de la Plana, Spain

**Keywords:** Astigmatism, Javal’s rule, Power vectors, Posterior astigmatism, Total corneal astigmatism

## Abstract

**Purpose:**

The scientific community has established Javal’s rule as a model linking refractive (RA) and keratometric (KA) astigmatism since its appearance more than 100 years ago. The aim was to improve the accuracy of this relationship according to subject’s age by applying the power vector analysis. Posterior corneal curvature has also been studied.

**Methods:**

The IOLMaster 700 optical biometer was used to measure the corneal thickness and the radius of curvature of the anterior and posterior corneal surfaces. Refractive error was determined by a non-cycloplegic subjective refraction process with trial lenses. Linear regression analyses were applied using *J*_0_ and *J*_45_ power vector components. An evaluation was carried out according to the subject’s age resulting into eight regression relationships for each astigmatic vector component for each relationship.

**Results:**

A total of 2254 right eyes from 2254 healthy subjects were evaluated. A trend towards against-the-rule astigmatism (ATR) was found with aging, both for refractive astigmatism (RA) and keratometric astigmatism (KA), with 95.2% of subjects under 20 years old having with-the-rule (WTR) KA, and only 22.8% above 79 years old. The following regression equations were found between RA and KA: ${J}_0^{\textrm{RA}}$ = 0.73 × ${J}_0^{\textrm{KA}}$ − 0.18 (*R* = 0.78) and ${J}_{45}^{\textrm{RA}}$ = 0.70 × ${J}_{45}^{\textrm{KA}}$ + 0.04 (*R* = 0.69) and between RA and total corneal astigmatism (TCA): ${J}_0^{\textrm{RA}}$ = 0.73 × ${J}_0^{\textrm{TCA}}$ + 0.13 (R=0.78) and ${J}_{45}^{\textrm{RA}}$ = 0.70 × ${J}_{45}^{\textrm{TCA}}$ − 0.06 (*R* = 0.68) for the whole sample, but with sensible differences among age groups, both in the slope and in the intercept.

**Conclusion:**

Ignoring the age of the subject when using Javal’s rule could lead to an error in the final cylinder calculation that would increase in high astigmatisms. Applying this new power vector approach based on subject’s age could improve the accuracy of the astigmatism prediction.


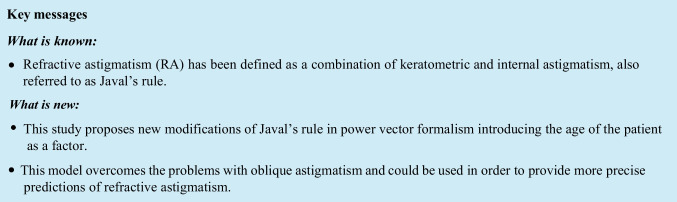


## Introduction

A proper determination of astigmatism is essential for many ophthalmological and optometric procedures, especially when they are irreversible, such as refractive corneal surgery or toric intraocular lens (IOL) implantation. Traditionally, the most of refractive astigmatism was assumed to be provided by the anterior surface of the cornea, omitting other refractive surfaces of the eye [1, 2]. Since the development of the first keratometers [3–5] the anterior astigmatism of the cornea has been extensively studied, resulting in the development of a linear relationship between refractive astigmatism (RA) and keratometric astigmatism (KA), known as Javal’s rule [4]. It should be noted that Javal, in 1890, proposed this relationship as an empirical estimation of the refractive astigmatism based on the anterior corneal surface, giving the anterior corneal power a weight of 1.25 and the internal astigmatism a cylinder of −0.50 D × 90° (RA = 1.25 × KA − 0.50 × 90°).

Although Javal’s rule is widely accepted, clinicians do have a simpler alternative which was suggested by Grosvenor et al. at the end of the 1980s [6]. They proposed a simplification of Javal’s rule concluding that, in with-the-rule (WTR) and against-the-rule (ATR) astigmatisms, anterior corneal weight in total astigmatism calculation was reduced to 1, resulting in the Javal’s rule simplification RA = KA − 0.50 × 90°. Recently, other authors [7–9] developed their own linear relationships using power vectors so as to avoid the limitations of Javal’s rule and Grosvenor’s approximation when corneal astigmatism is not exactly oriented at 0° or 90° or astigmatism higher than 2.00 D. All these studies resolved Grosvenor’s weaknesses, resulting in two formulas, one for each astigmatic power vector component. However, all those studies only incorporate data from the anterior corneal surface while posterior corneal astigmatism was completely neglected.

Historically, posterior corneal astigmatism was believed to be irrelevant, even, for example, in those cases where calculations for toric IOL surgery were made [10] Nevertheless, some studies have shown an astigmatic miscorrection after crystalline lens replacement by toric IOLs, even when calculation formulas were properly used [11–14] Early attempts to define the magnitude of posterior corneal astigmatism were more primitive and relied on fewer and pre-defined points of the cornea [15–17] Subsequent studies have used more sophisticated systems such as rotating Scheimpflug imaging [18–22] and optical coherence tomography (OCT) [23, 24]. By measuring the astigmatism of both corneal surfaces and considering the corneal thickness, these studies have been able to calculate the total corneal astigmatism and compare it to the keratometric astigmatism, uncovering a source of error in previous calculations.

When discussing astigmatism, a crucial factor must be taken into account: the age of the subject. Previous studies [18, 25, 26] have reported changes in the axis orientation of refractive and corneal astigmatism produced over the years.

The purpose of the current study was to develop a new linear regression model between corneal and refractive astigmatisms by dependent on the age of the subject.

## Materials and methods

### Subjects

A total of 2254 right eyes from 2254 Caucasian healthy subjects were included in this transversal and retrospective study. To avoid bias, only data from the right eye were selected [27].

Data were collected from subjects who had undergone ophthalmological examinations in an ophthalmological center in Spain between 2017 and 2020. Written informed consent was obtained in each case and the tenets of the Declaration of Helsinki were followed. Subjects over 7 years old were included unless they presented any of the following exclusion criteria: bad quality measurement, any type of ocular disease, previous ocular surgery, contact lens wearers within 1 week before the data acquisition, or no data from the right eye. The subjects were divided into eight age groups i.e. under 20, over 79, and every decade between 20 and 79 years old.

### Data collection

Demographic and ocular data were collected including age, gender, and refractive and corneal astigmatism. Refractive astigmatism was based on non-cycloplegic subjective refraction obtained by using trial frame and trial lenses. The distance vertex was set to 12 mm and distometry was applied to convert the refractive power at the trial frame’s plane to the corrected power at the cornea’s plane, thus enabling the direct comparison of refractive and corneal results.

Radii from principal meridians of both corneal surfaces and corneal thickness were obtained using an OCT-based device with a telecentric keratometer system (IOLMaster 700, Carl Zeiss Meditec, Germany). The IOLMaster 700 is an optical biometer based on swept-source OCT that provides high-resolution and high-speed scans of the cornea. It has been shown that IOLMaster 700 has a good repeatability of the anterior and posterior corneal powers [24, 28, 29].

Measurements provided by the IOLMaster 700 were directly achieved as the corneal radius expressed in millimeters and were used to compute corneal astigmatism in the form of:

Keratometric astigmatism (KA): Expressed as astigmatism provided by the anterior surface of the cornea when corneal power is calculated for 1.3375 keratometric refractive index value:

1 $$\textrm{KA}=\frac{1.3375-1}{r_{\textrm{s}}^{\textrm{AC}}}-\frac{1.3375-1}{r_{\textrm{f}}^{\textrm{AC}}}$$

where ${r}_{\textrm{s}}^{\textrm{AC}}$ refers to the steepest anterior cornea meridian and ${r}_{\textrm{f}}^{\textrm{AC}}$ refers to the flattest one.

Anterior corneal astigmatism (ACA): expressed as astigmatism provided by the anterior surface of the cornea when corneal power is calculated for 1.376 stroma refractive index value:

2 $$\textrm{ACA}=\frac{1.376-1}{r_{\textrm{s}}^{\textrm{AC}}}-\frac{1.376-1}{r_{\textrm{f}}^{\textrm{AC}}}$$

Posterior corneal astigmatism (PCA): expressed as astigmatism provided by the posterior surface of the cornea, considering corneal stroma (1.376) and aqueous humor (1.336) refractive indexes:

3 $$\textrm{PCA}=\frac{1.336-1.376}{r_{\textrm{s}}^{\textrm{PC}}}-\frac{1.336-1.376}{r_{\textrm{f}}^{\textrm{PC}}}$$

where ${r}_{\textrm{s}}^{\textrm{PC}}$ refers to the steepest posterior cornea meridian and ${r}_{\textrm{f}}^{\textrm{PC}}$ refers to the flattest one.

Total corneal astigmatism (TCA): Expressed as astigmatism provided by the anterior and the posterior surface of the cornea considering the corneal thickness (*t*) when principal meridians of both surfaces coincide:

4 $$\textrm{TCA}=\textrm{ACA}+\textrm{PCA}-\frac{t}{1.376}\times \textrm{ACA}\times \textrm{PCA}$$

when axis astigmatism are not the same, calculations are made in vectorial form as it is described further on.

The RA, KA, and ACA were classified into three groups: eyes with with-the-rule (WTR) astigmatism, where the steepest meridian is vertical or within 30° of the 90° meridian, eyes with against-the-rule (ATR) astigmatism, where the steepest meridian was between 0° ± 30°, and oblique in the remaining cases [30]. In order to avoid confusion and misunderstanding, the classical terms WTR and ATR are not used when discussing PCA due to the negative power values obtained from the refractive index, and because those “rules” have been classically described for the anterior corneal astigmatism classification, but not for the posterior.

### Data analysis

The following equations were used to convert main power meridians into power vector notation as described by Thibos et al. [31] to conduct the appropriate calculations and comparisons between refractive and corneal astigmatisms.

5 $${J}_0=-\frac{P_{\textrm{s}}-{P}_{\textrm{f}}}{2}\cos 2\upbeta$$

6 $${J}_{45}=-\frac{P_{\textrm{s}}-{P}_{\textrm{f}}}{2}\sin 2\upbeta$$

where *P*_s_ was the power of the steepest meridian, *P*_f_ was the power of the flattest meridian and *β* was the orientation of the steepest meridian. Equations 5 and 6 were applied for RA, KA, ACA, PCA, and TCA.

Statistical analysis was performed using the SPSS software v.24.0 for Windows (IBM Corporation, USA). The linear regression adjustment was employed to study the relationship between the TCA vs ACA, PCA vs ACA, RA vs TCA, and RA vs KA, and Pearson correlation coefficient (*R*) is calculated for each case.

## Results

**Fig. 1:**
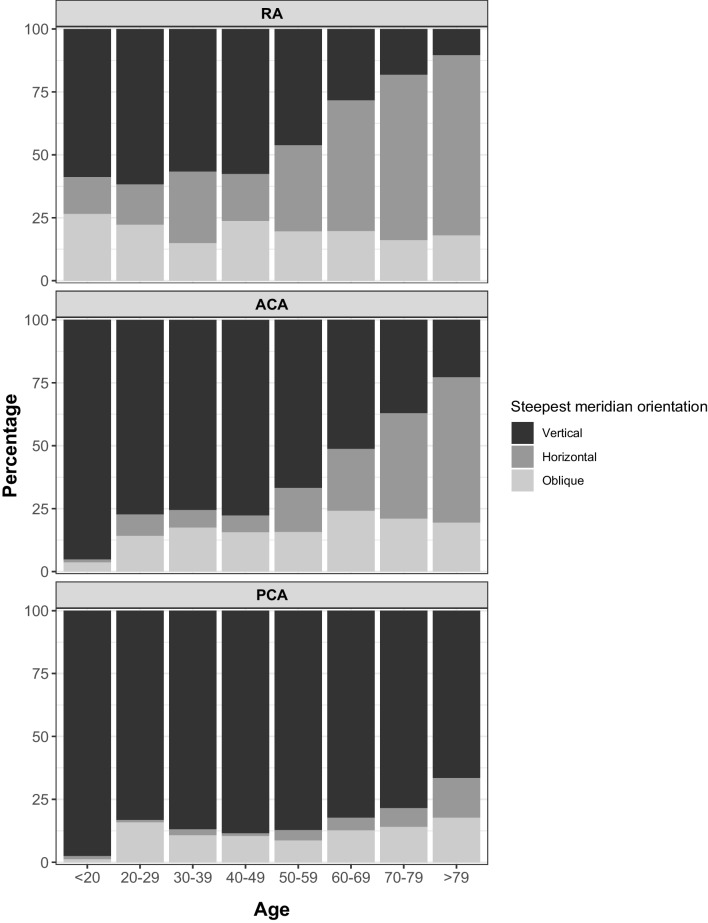
Distribution of eyes by age groups for refractive, anterior corneal, and posterior corneal astigmatism in function of the steepest meridian orientation

The statistics for refractive values and keratometric, anterior corneal, posterior corneal and total corneal astigmatism by age groups are summarized in Table 1. The distribution of eyes by age groups for refractive, anterior cornea, and posterior cornea astigmatism in function the steepest meridian is showed in Fig. 1. There was a strong change in the steepest meridian in ACA decreasing from 95.2% of the cases with vertical steepest meridian in subjects under 20 years to only the 22.8% in subjects over 79 years.

**Table 1:** Sample statistics for refractive values and keratometric, anterior corneal, posterior corneal and total corneal astigmatism by age groups

Age group	*N*	Age (years) mean ± SD (min, max)	Female Gender *n*; (%)	SE_C_ (*D*) mean ± SD (min[-], max[+])	Cyl_C_ (*D*) mean ± SD (min, max)	KA (*D*) mean ± SD (min, max)	ACA (*D*) mean ± SD (min, max)	PCA (*D*) mean ± SD (min, max)	TCA (*D*) mean ± SD (min, max)
Total sample	2254	64 ± 18 (7, 96)	1356; 60%	−1.02 ± 3.85 (−20.53, + 8.85)	−0.81 ± 0.80 (0, −6.04)	−0.94 ± 0.70 (0, −5.50)	−1.04 ± 0.78 (0, −6.13)	+0.21 ± 0.13 (+0.92, 0.00)	−0.94 ± 0.69 (−0.05, −5.64)
< 20	83	13 ± 3 (7, 18)	49; 59%	−1.88 ± 2.16 (−7.60, + 3.65)	−0.36 ± 0.58 (0, −3.04)	−0.97 ± 0.51 (−0.20, −2.91)	−1.08 ± 0.57 (−0.23, −3.25)	+0.22 ± 0.09 (+0.44, +0.06)	−0.89 ± 0.50 (−0.08, −2.82)
20–29	108	26 ± 2 (20, 29)	59; 55%	−5.18 ± 3.09 (−15.00, +4.91)	−0.72 ± 0.66 (0, −3.69)	−1.04 ± 0.58 (0, −2.41)	−1.16 ± 0.64 (0, −2.69)	+0.22 ± 0.10 (+0.50, 0.00)	−0.97 ± 0.54 (−0.13, −2.27)
30–39	87	34 ± 3 (30, 39)	52; 60%	−5.21 ± 3.30 (−16.50, + 7.21)	−0.80 ± 0.87 (0, −4.95)	−0.99 ± 0.75 (0, −4.24)	−1.11 ± 0.84 (0, −4.72)	+0.21 ± 0.13 (+0.72, 0.00)	−0.93 ± 0.71 (−0.12, −4.14)
40–49	90	45 ± 3 (40, 49)	47; 52%	−4.08 ± 5.30 (−20.53, +7.20)	−0.76 ± 0.85 (0, −3.67)	−1.08 ± 0.82 (−0.13, −5.50)	−1.20 ± 0.92 (−0.15, −6.13)	+0.26 ± 0.15 (+0.92, 0.00)	−0.98 ± 0.81 (−0.07, −5.64)
50–59	223	55 ± 3 (50, 59)	128; 57%	−1.55 ± 5.02 (−19.80, +8.70)	−0.77 ± 0.83 (0, −4.36)	−1.02 ± 0.65 (−0.12, −3.59)	−1.13 ± 0.72 (−0.13, −4.00)	+0.25 ± 0.13 (+0.68, 0.00)	−0.95 ± 0.62 (−0.08, −3.47)
60–69	618	65 ± 3 (60, 69)	386; 63%	−0.74 ± 3.94 (−19.80, +8.85)	−0.78 ± 0.78 (0, −6.04)	−0.90 ± 0.72 (0, −5.09)	−1.00 ± 0.8 (0, −5.67)	+0.22 ± 0.12 (+0.74, 0.00)	−0.89 ± 0.70 (−0.05, −4.95)
70–79	810	74 ± 3 (70, 79)	496; 61%	−0.03 ± 2.87 (−18.00, +7.64)	−0.86 ± 0.78 (0, −5.80)	−0.88 ± 0.69 (0, −5.32)	−0.98 ± 0.76 (0, −5.92)	+0.20 ± 0.13 (+0.90, 0.00)	−0.91 ± 0.67 (−0.05, −5.02)
> 79	235	84 ± 4 (80, 96)	139; 59%	+0.26 ± 2.26 (−9.68, +5.89)	−0.96 ± 0.86 (0, −5.93)	−1.04 ± 0.75 (0, −4.80)	−1.16 ± 0.83 (0, −5.34)	+0.18 ± 0.12 (+0.75, 0.00)	−1.12 ± 0.77 (−0.06, −4.60)

*SD* standard deviation, *SE*_C_ spherical equivalent in corneal plane, *Cyl*_C_ cylinder in corneal plane, *KA* keratometric astigmatism, *ACA* anterior corneal astigmatism, *PCA* posterior corneal astigmatism, *TCA* total corneal astigmatism

Mean values of RA, KA, ACA, PCA, and TCA are summarized in Table 2 in terms of power vectors with the standard deviation and the maximum and the minimum values, both for the whole sample, and for each age group. The RA, KA, and ACA show a clear trend in *J*_0_ power vector component from positive values under 60 years old to negative values above 60 years old, this being related with the trend in corneal astigmatism from WTR to ATR as we age. The behavior of the PCA power vector *J*_0_ component is constant, maintaining negative values in all age groups.

**Table 2:** Mean values of refractive and corneal astigmatism.

	J_0_					J_45_				
Age group	RA mean ± SD (min, max)	KA mean ± SD (min, max)	ACA mean ± SD (min, max)	PCA mean ± SD (min, max)	TCA mean ± SD (min, max)	RA mean ± SD (min, max)	KA mean ± SD (min, max)	ACA mean ± SD (min, max)	PCA mean ± SD (min, max)	TCA mean ± SD (min, max)
Total sample	-0.10 ± 0.47 (-2.31, +3.00)	+0.11 ± 0.49 (-1.88, +2.63)	+0.12 ± 0.55 (-2.10, +2.93)	-0.08 ± 0.07 (-0.43, +0.32)	+0.04 ± 0.50 (-1.89, +2.60)	+0.02 ± 0.30 (-1.87, +2.51)	-0.02 ± 0.29 (-1.63, +1.77)	-0.02 ± 0.33 (-1.81, +1.97)	-0.03 ± 0.05 (-0.45, +0.22)	-0.05 ± 0.29 (-1.62, +1.52)
<20	+0.09 ± 0.27 (-0.46, +1.43)	+0.45 ± 0.26 (-0.06, +1.44)	+0.50 ± 0.29 (-0.07, +1.61)	-0.10 ± 0.05 (-0.22, +0.03)	+0.40 ± 0.26 (-0.15, +1.39)	-0.03 ± 0.19 (-0.90, +0.71)	+0.00 ± 0.18 (-0.69, +0.52)	+0.00 ± 0.21 (-0.76, +0.58)	-0.03 ± 0.04 (-0.14, +0.07)	-0.02 ± 0.18 (-0.73, +0.44)
20-29	+0.11 ± 0.38 (-1.41, +1.01)	+0.37 ± 0.38 (-0.86, +1.20)	+0.41 ± 0.42 (-0.95, +1.33)	-0.09 ± 0.06 (-0.25, +0.11)	+0.32 ± 0.37 (-0.88, +1.12)	+0.04 ± 0.28 (-0.78, +1.19)	+0.03 ± 0.27 (-0.87, +0.88)	+0.04 ± 0.30 (-0.97, +0.98)	-0.03 ± 0.05 (-0.19, +0.08)	+0.01 ± 0.27 (-0.93, +0.79)
30-39	+0.13 ± 0.51 (-1.59, +2.44)	+0.35 ± 0.46 (-0.93, +2.11)	+0.39 ± 0.51 (-1.04, +2.36)	-0.09 ± 0.07 (-0.36, +0.09)	+0.30 ± 0.45 (-0.95, +2.06)	+0.03 ± 0.26 (-1.02, +0.69)	+0.02 ± 0.24 (-0.74, +0.60)	+0.02 ± 0.26 (-0.82, +0.67)	-0.03 ± 0.04 (-0.12, +0.08)	-0.01 ± 0.24 (-0.74, +0.57)
40-49	+0.14 ± 0.40 (-0.97, +1.40)	+0.34 ± 0.47 (-1.36, +2.63)	+0.37 ± 0.51 (-1.51, +2.93)	-0.10 ± 0.07 (-0.34, +0.17)	+0.27 ± 0.45 (-1.34, +2.60)	+0.14 ± 0.36 (-0.54, +1.48)	+0.11 ± 0.35 (-0.49, +1.77)	+0.12 ± 0.39 (-0.55, +1.97)	-0.04 ± 0.07 (-0.45, +0.21)	+0.08 ± 0.34 (-0.54, +1.52)
50-59	+0.04 ± 0.46 (-1.92, +2.15)	+0.29 ± 0.44 (-1.12, +1.80)	+0.32 ± 0.49 (-1.25, +2.00)	-0.10 ± 0.07 (-0.34, +0.15)	+0.21 ± 0.43 (-1.20, +1.74)	+0.04 ± 0.33 (-1.15, +1.90)	+0.01 ± 0.30 (-1.06, +1.32)	+0.02 ± 0.34 (-1.18, +1.48)	-0.04 ± 0.05 (-0.26, +0.14)	-0.02 ± 0.30 (-1.09, +1.26)
60-69	-0.06 ± 0.46 (-1.53, +3.00)	+0.16 ± 0.48 (-1.59, +2.49)	+0.18 ± 0.53 (-1.77, +2.78)	-0.09 ± 0.07 (-0.37, +0.32)	+0.10 ± 0.48 (-1.62, +2.41)	+0.02 ± 0.29 (-1.13, +2.04)	-0.01 ± 0.29 (-1.40, +1.45)	-0.01 ± 0.32 (-1.56, +1.62)	-0.03 ± 0.05 (-0.26, +0.12)	-0.04 ± 0.29 (-1.44, +1.36)
70-79	-0.19 ± 0.46 (-2.31, +2.59)	-0.01 ± 0.47 (-1.88, +2.60)	-0.01 ± 0.52 (-2.10, +2.90)	-0.07 ± 0.07 (-0.43, +0.20)	-0.08 ± 0.47 (-1.89, +2.46)	+0.16 ± 0.30 (-1.87, +2.51)	-0.06 ± 0.29 (-1.63, +1.40)	-0.06 ± 0.33 (-1.81, +1.56)	-0.03 ± 0.05 (-0.24, +0.22)	-0.09 ± 0.29 (-1.62, +1.33)
>79	-0.31 ± 0.45 (-1.62, +1.91)	-0.21 ± 0.51 (-1.70, +2.32)	-0.23 ± 0.56 (-1.89, +2.58)	-0.05 ± 0.07 (-0.36, +0.11)	-0.28 ± 0.51 (-1.88, +2.22)	+0.03 ± 0.34 (-1.55, +2.27)	-0.05 ± 0.33 (-1.54, +1.50)	-0.06 ± 0.37 (-1.72, +1.67)	-0.03 ± 0.05 (-0.26, +0.20)	-0.09 ± 0.33 (-1.52, +1.41)

SD: standard deviation; RA: refractive astigmatism; KA: keratometric astigmatism; ACA: anterior corneal astigmatism; PCA: posterior corneal astigmatism; TCA: total corneal astigmatism. J_0_>0 WTR astigmatism; J_0_<0 ATR astigmatism (In PCA values not applied rule)

**Fig. 2:**
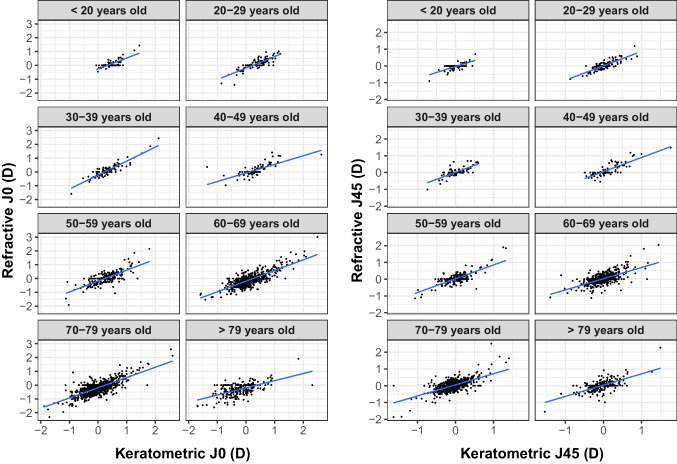
Scatterplots (black) and linear regression lines (blue) between refractive and keratometric astigmatisms for *J*_0_ and *J*_45_ astigmatic power-vector components by age group

Regression equations and Pearson correlation coefficients (*R*) by age group for TCA vs ACA, PCA vs ACA, RA vs TCA, and RA vs KA are summarized in Table 3A, B, C and D, respectively. Fig. 2 shows the scatter diagrams of the *J*_0_ and *J*_45_ component of refractive and keratometric astigmatism for each age group, that is, the Javal’s rule in power vector components for age groups.

**Table 3:** Regression equations and Pearson correlation coefficients (R) by age group of total cornea vs anterior corneal astigmatism (A), posterior cornea vs anterior cornea astigmatism (B), refractive vs total cornea astigmatism (C), and refractive vs keratometric astigmatism (D)

A	TCA vs ACA			
	*J*_0_		*J*_45_	
	*R*	Equation	*R*	Equation
Total sample	0.99	${J}_0^{\textrm{TCA}}$ = 0.90 × ${J}_0^{\textrm{ACA}}$ − 0.07	0.99	${J}_{45}^{\textrm{TCA}}$ = 0.89 × ${J}_{45}^{\textrm{ACA}}$ − 0.03
< 20	0.99	${J}_0^{\textrm{TCA}}$ = 0.89 × ${J}_0^{\textrm{ACA}}$ − 0.05	0.99	${J}_{45}^{\textrm{TCA}}$ = 0.87 × ${J}_{45}^{\textrm{ACA}}$ − 0.03
20–29	0.99	${J}_0^{\textrm{TCA}}$ = 0.88 × ${J}_0^{\textrm{ACA}}$ − 0.04	0.99	${J}_{45}^{\textrm{TCA}}$ = 0.88 × ${J}_{45}^{\textrm{ACA}}$ − 0.02
30–39	0.99	${J}_0^{\textrm{TCA}}$ = 0.87 × ${J}_0^{\textrm{ACA}}$ − 0.04	0.99	${J}_{45}^{\textrm{TCA}}$ = 0.89 × ${J}_{45}^{\textrm{ACA}}$ − 0.03
40–49	0.99	${J}_0^{\textrm{TCA}}$= 0.88 × ${J}_0^{\textrm{ACA}}$ − 0.06	0.99	${J}_{45}^{\textrm{TCA}}$ = 0.86 × ${J}_{45}^{\textrm{ACA}}$ − 0.02
50–59	0.99	${J}_0^{\textrm{TCA}}$ = 0.88 × ${J}_0^{\textrm{ACA}}$ − 0.06	0.99	${J}_{45}^{\textrm{TCA}}$ = 0.88 × ${J}_{45}^{\textrm{ACA}}$ − 0.03
60–69	0.99	${J}_0^{\textrm{TCA}}$ = 0.90 × ${J}_0^{\textrm{ACA}}$ − 0.07	0.99	${J}_{45}^{\textrm{TCA}}$ = 0.90 × ${J}_{45}^{\textrm{ACA}}$ − 0.03
70–79	0.99	${J}_0^{\textrm{TCA}}$ = 0.89 × ${J}_0^{\textrm{ACA}}$ − 0.08	0.99	${J}_{45}^{\textrm{TCA}}$ = 0.89 × ${J}_{45}^{\textrm{ACA}}$ − 0.04
> 79	0.99	${J}_0^{\textrm{TCA}}$ = 0.91 × ${J}_0^{\textrm{ACA}}$ − 0.07	0.99	${J}_{45}^{\textrm{TCA}}$ = 0.90 × ${J}_{45}^{\textrm{ACA}}$ − 0.04
B	PCA vs ACA			
	***J***_**0**_		***J***_**45**_	
	***R***	Equation	***R***	Equation
Total sample	0.77	${J}_0^{\textrm{PCA}}$ = − 0.10 × ${J}_0^{\textrm{ACA}}$ − 0.07	0.71	${J}_{45}^{\textrm{PCA}}$ = − 0.11 × ${J}_{45}^{\textrm{ACA}}$ − 0.03
< 20	0.73	${J}_0^{\textrm{PCA}}$ = − 0.11 × ${J}_0^{\textrm{ACA}}$ − 0.05	0.76	${J}_{45}^{\textrm{PCA}}$ = − 0.13 × ${J}_{45}^{\textrm{ACA}}$ − 0.03
20–29	0.84	${J}_0^{\textrm{PCA}}$ = − 0.12 × ${J}_0^{\textrm{ACA}}$ − 0.04	0.81	${J}_{45}^{\textrm{PCA}}$ = − 0.12 × ${J}_{45}^{\textrm{ACA}}$ − 0.02
30–39	0.89	${J}_0^{\textrm{PCA}}$ = − 0.13 × ${J}_0^{\textrm{ACA}}$ − 0.04	0.74	${J}_{45}^{\textrm{PCA}}$ = − 0.11 × ${J}_{45}^{\textrm{ACA}}$ − 0.03
40–49	0.85	${J}_0^{\textrm{PCA}}$ = − 0.12 × ${J}_0^{\textrm{ACA}}$ − 0.06	0.76	${J}_{45}^{\textrm{PCA}}$ = − 0.14 × ${J}_{45}^{\textrm{ACA}}$ − 0.02
50–59	0.79	${J}_0^{\textrm{PCA}}$ = − 0.12 × ${J}_0^{\textrm{ACA}}$ − 0.06	0.79	${J}_{45}^{\textrm{PCA}}$ = − 0.12 × ${J}_{45}^{\textrm{ACA}}$ − 0.03
60–69	0.75	${J}_0^{\textrm{PCA}}$ = − 0.10 × ${J}_0^{\textrm{ACA}}$ − 0.07	0.69	${J}_{45}^{\textrm{PCA}}$ = − 0.10 × ${J}_{45}^{\textrm{ACA}}$ − 0.03
70–79	0.76	${J}_0^{\textrm{PCA}}$ = − 0.11 × ${J}_0^{\textrm{ACA}}$ − 0.08	0.71	${J}_{45}^{\textrm{PCA}}$ = − 0.11 × ${J}_{45}^{\textrm{ACA}}$ − 0.04
> 79	0.74	${J}_0^{\textrm{PCA}}$ = − 0.10 × ${J}_0^{\textrm{ACA}}$ − 0.07	0.69	${J}_{45}^{\textrm{PCA}}$ = − 0.10 × ${J}_{45}^{\textrm{ACA}}$ − 0.04
C	RA vs TCA			
	*J*_0_		*J*_45_	
	*R*	Equation	*R*	Equation
Total sample	0.78	${J}_0^{\textrm{RA}}$ = 0.73 × ${J}_0^{\textrm{TCA}}$+ 0.13	0.68	${J}_{45}^{\textrm{RA}}$ = 0.70 × ${J}_{45}^{\textrm{TCA}}$ − 0.06
< 20	0.78	${J}_0^{\textrm{RA}}$ = 0.79 × ${J}_0^{\textrm{TCA}}$ − 0.22	0.69	${J}_{45}^{\textrm{RA}}$ = 0.73 × ${J}_{45}^{\textrm{TCA}}$ − 0.01
20–29	0.83	${J}_0^{\textrm{RA}}$ = 0.86 × ${J}_0^{\textrm{TCA}}$ − 0.16	0.84	${J}_{45}^{\textrm{RA}}$ = 0.87 × ${J}_{45}^{\textrm{TCA}}$ + 0.03
30–39	0.91	${J}_0^{\textrm{RA}}$ = 1.00 × ${J}_0^{\textrm{TCA}}$ − 0.18	0.74	${J}_{45}^{\textrm{RA}}$ = 0.83 × ${J}_{45}^{\textrm{TCA}}$ + 0.03
40–49	0.72	${J}_0^{\textrm{RA}}$ = 0.63 × ${J}_0^{\textrm{TCA}}$ - 0.04	0.84	${J}_{45}^{\textrm{RA}}$ = 0.88 × ${J}_{45}^{\textrm{TCA}}$ + 0.08
50–59	0.76	${J}_0^{\textrm{RA}}$ = 0.81 × ${J}_0^{\textrm{TCA}}$ - 0.13	0.78	${J}_{45}^{\textrm{RA}}$ = 0.84 × ${J}_{45}^{\textrm{TCA}}$ + 0.05
60–69	0.79	${J}_0^{\textrm{RA}}$ = 0.77 × ${J}_0^{\textrm{TCA}}$ + 0.13	0.64	${J}_{45}^{\textrm{RA}}$ = 0.66 × ${J}_{45}^{\textrm{TCA}}$ + 0.05
70–79	0.76	${J}_0^{\textrm{RA}}$ = 0.74 × ${J}_0^{\textrm{TCA}}$ − 0.13	0.64	${J}_{45}^{\textrm{RA}}$ = 0.66 $\times {J}_{45}^{\textrm{TCA}}$ + 0.08
> 79	0.58	${J}_0^{\textrm{RA}}$ = 0.51 × ${J}_0^{\textrm{TCA}}$ − 0.16	0.63	${J}_{45}^{\textrm{RA}}$ = 0.65 × ${J}_{45}^{\textrm{TCA}}$ + 0.06
D	RA vs KA			
	*J*_0_		*J*_45_	
	*R*	Equation	*R*	Equation
Total sample	0.78	${J}_0^{\textrm{RA}}$ = 0.73 × ${\textrm{J}}_0^{\textrm{KA}}$ − 0.18	0.69	${J}_{45}^{\textrm{RA}}$ = 0.70 × ${J}_{45}^{\textrm{KA}}$ + 0.04
< 20	0.78	${J}_0^{\textrm{RA}}$ = 0.79 × ${\textrm{J}}_0^{\textrm{KA}}$ − 0.26	0.70	${J}_{45}^{\textrm{RA}}$ = 0.72 × ${J}_{45}^{\textrm{KA}}$ −0.03
20–29	0.82	${J}_0^{\textrm{RA}}$ = 0.84 × ${\textrm{J}}_0^{\textrm{KA}}$ − 0.20	0.84	${J}_{45}^{\textrm{RA}}$ = 0.86 × ${J}_{45}^{\textrm{KA}}$ + 0.01
30–39	0.91	${J}_0^{\textrm{RA}}$ = 1.00 × ${\textrm{J}}_0^{\textrm{KA}}$ − 0.23	0.75	${J}_{45}^{\textrm{RA}}$ = 0.84 × ${J}_{45}^{\textrm{KA}}$ + 0.01
40–49	0.72	${J}_0^{\textrm{RA}}$ = 0.62 × ${J}_0^{\textrm{KA}}$ − 0.07	0.83	${J}_{45}^{\textrm{RA}}$ = 0.84 × ${J}_{45}^{\textrm{KA}}$ + 0.06
50–59	076	${J}_0^{\textrm{RA}}$ = 0.79 × ${J}_0^{\textrm{KA}}$ − 0.18	0.77	${J}_{45}^{\textrm{RA}}$ = 0.82 × ${J}_{45}^{\textrm{KA}}$ + 0.02
60–69	0.79	${J}_0^{\textrm{RA}}$ = 0.77 × ${J}_0^{\textrm{KA}}$ − 0.19	0.65	${J}_{45}^{\textrm{RA}}$ = 0.67 × ${J}_{45}^{\textrm{KA}}$ + 0.02
70–79	0.76	${J}_0^{\textrm{RA}}$ = 0.74 × ${J}_0^{\textrm{KA}}$ − 0.19	0.65	${J}_{45}^{\textrm{RA}}$ = 0.66 × ${J}_{45}^{\textrm{KA}}$ + 0.05
> 79	0.58	${J}_0^{\textrm{RA}}$ = 0.52 × ${J}_0^{\textrm{KA}}$ − 0.20	0.65	${J}_{45}^{\textrm{RA}}$ = 0.67 × ${J}_{45}^{\textrm{KA}}$ + 0.04

*R* Pearson correlation coefficient, *RA* refractive astigmatism, *KA* keratometric astigmatism, *ACA* anterior corneal astigmatism, *PCA* posterior corneal astigmatism, *TCA* total corneal astigmatism

According to the correlation coefficient, TCA and ACA are highly correlated in all age groups studied and for both astigmatism components, *J*_0_ and *J*_45_ (*R* = 0.99 in all cases summarized in table 3A).

The slope (multiplicand) in the linear regression equations defines the weight of one regressor (*x*) as a predictor of the dependent variable (*y*), and in the case of the relation between PCA vs ACA ( ${J}_0^{\textrm{PCA}}$ = − 0.10 × ${J}_0^{\textrm{ACA}}$ − 0.07) , this is the lowest with values of −0.10, against the highest values in equations found for RA vs TCA (${J}_0^{\textrm{RA}}$ = 1.00 × ${J}_0^{\textrm{TCA}}$ − 0.18) and RA vs KA (${J}_0^{\textrm{RA}}$ = 1.00 × ${\textrm{J}}_0^{\textrm{KA}}$ − 0.23).

General equations have been obtained considering the whole sample and for each group of age resulting in a suitable Javal’s rule depending on subject’s age.

## Discussion

The present study had been designed to develop new linear relationships between corneal and refractive astigmatism according to the age of the subject. At the same time, authors assessed the peculiarities of corneal astigmatism, allowing for the analysis between astigmatism from both surfaces of the cornea, as well as total corneal astigmatism. The large sample size has enabled an improved characterization of the demographic aspects of astigmatism prevalence and its axis orientation.

Previous investigations have shown that astigmatism orientation does not remain stable over the years [26, 32]. Both the ACA and PCA in the current study showed a trend of steepest meridian location towards a more horizontal orientation with increasing age, more evident for ACA than PCA (see Fig. 1).

Regarding ACA, current results have shown an increase in the cases of eyes with ATR from the age of 50 (see Fig. 1), which coincides with the increment demonstrated by previous studies [19–22, 26, 32], keeping the cases of oblique orientation changing without a well-defined pattern, even though it seemed that there is a small rise of the percentage in senior subjects [19–21, 23, 32]. In contrast, for PCA, the vast majority of the studied sample showed a steepest vertical meridian, with a slight increase in the percentage of eyes with steepest horizontal meridian after the 7th decade (see Fig. 1). However, *J*_0_ power-vector component values remained negative over the age groups (see Table 2). This result is in agreement with other authors [19–23, 32, 33]

The TCA had a similar behavior to the ACA, producing a change from WTR to ATR from the age of 70. Nasser et al. observed this change in older subjects, at the age of 80 [26].

We have found an increase in the percentage of eyes with ATR-RA (*J*_0_ < 0) over WTR-RA (*J*_0_ > 0) above the 5th/6th decades (see Fig. 1) accompanied by a change from positive to negative *J*_0_ values in both ACA and KA from the age of 70 (see Table 1), those findings agreeing with other studies [19–22, 26, 32].

Regarding the relation between TCA and ACA, there is a strong correlation between both parameters with a Pearson correlation coefficient almost equal to 1 for all age groups, thus suggesting that the changes in anterior corneal surface would highly affect TCA. The slope values in those linear regression equations, below 1.00 but with an almost constant value all over the age groups (between 0.87 and 0.91 for *J*_0_ and between 0.87 and 0.90 for *J*_0_) point to an almost constant contribution of PCA to TCA. In this line, table 2 shows PCA *J*_0_ and PCA *J*_45_ values of −0.08 ± 0.07 D and −0.03 ± 0.05 D respectively for the whole sample, implying that around 0.15 D of cylinder is not considered when only the first surface of the cornea is measured. This estimation error of RA based only on KA instead of TCA can change related to the subject’s age, as can be seen in Table 2 (with *J*_0_ PCA values changing from −0.10 D for the < 20 years old group to −0.05 D for the > 79 years old group). Previous studies concluded that around a 0.25 D of astigmatism is underestimated when KA is employed instead of TCA [19, 20, 22, 24], but those studies did not consider the age.

Current results demonstrate a linear relationship between PCA and ACA, where PCA tended to be “−0.1 × ACA” for *J*_0_ as well as *J*_45_ and remained quite stable over the years showing a high correlation, even though the better correlation coefficients are obtained between the 2nd and 5th decades (see Table 3B).

To the best of our knowledge, this is the first study attempting to examine the relationship between refractive and keratometric astigmatisms dividing the sample into eight different age groups. This allows us to obtain a more detailed relationship based on age. As can be seen, the slope values for *J*_0_ range from 0.52 to 1.00, depending on the age group, while the constant value changes between −0.07 D and −0.23 D. This should be critical, since not tailoring the regression lines to the age of the subject could result in clinically significant misestimations of RA. For example, if the *J*_0_ power-vector component for KA is = 1.5 D, the prediction of *J*_0_ power-vector component RA could vary up to 0.5 D depending on the regression line used, resulting in a difference in cylinder power of 1 D. Therefore, disregarding these modifications by using a generic expression independent of age could result in a systematic error, as it has been observed in this study that the relationship between the refractive astigmatism, corneal astigmatism, and internal astigmatism varies with age. When making irreversible decisions, such as the implantation of a toric intraocular lens in refractive surgery, these age-related changes should be considered, especially when surgeries are performed in patients in their 50s or 60s. In relation to these results, and following the notation traditionally used in clinical practice, we could obtain the linear relationships between the KA and RA in terms of cylinder and axis. For the result obtained by considering the whole sample, the approximate linear relationship, obtained by averaging the slopes of the vector components, would be RA = 0.715 KA − 0.37 × 84°. It is important to note that vector notation is advantageous over conventional notation since it is the only one that allows operations and predictions to be made when corneal and internal astigmatisms do not have the same orientation. In order to allow comparisons between the present study and the other previously published, similar conditions were replicated in terms of distometry and age of the participants. Table 4 contains the regression equations provided by other authors and current simulated regressions equation for concretely each comparison in the same notation and under the same conditions. Tong et al. [7] evaluated astigmatism using the Retinomax K-Plus (Nikon Inc., Melville, NY) in children between 7 and 13 years, obtaining a good correlation for *J*_0_ and a moderate correlation for *J*_45_. In our study, similar results were found, with values of the slope and the constant being similar in both studies, particularly for *J*_0_. On the other hand, Remón et al. [8] conducted their study on adults under the age of 59, using the Nidek ARK 2000 Auto Ref/Keratometer resulting in a high correlation for *J*_0_ and *J*_45_, while the slope for *J*_0_ regression tended to 1 whereas slope for *J*_45_ was 1.46. The current study demonstrates lower slope values in both cases and a constant value for *J*_0_ inferior in 0.1 D. These slight differences cannot be attributed to the subjects’ racial backgrounds since both studies were conducted in Spain. Rather, they can be linked to the sample size; the present study includes 827 right eyes, while Remón et al. only had 105 right eyes, thus the larger sample size may produce more precise results. In addition, Remón’s study used an objective method to measure refraction, without taking into account whether it coincided with the subjective refraction.

**Table 4:** Regression equations between refractive and keratometric astigmatism obtained in previous studies and in the current study

			*J*_0_		*J*_45_	
*N*	Age (years)	Study	*R*	Equation	*R*	Equation
993	7 − 13 (7.7 ± 0.8)	Tong et al.	0.83	${J}_0^{\textrm{RA}}$ = 0.93 × ${J}_0^{\textrm{KA}}$ − 0.28	0.70	${J}_{45}^{\textrm{RA}}$ = 0.64 × ${J}_{45}^{\textrm{KA}}$ − 0.01
48	7 − 13 (10.9 ± 1.7)	Current study	0.81	${J}_0^{\textrm{RA}}$ = 0.91 × ${J}_0^{\textrm{KA}}$ − 0.30	0.62	${J}_{45}^{\textrm{RA}}$ = 0.77 × ${J}_{45}^{\textrm{KA}}$ − 0.04
105	18 − 59 (35 ± 9)	Remón et al.	0.89	${J}_0^{\textrm{RA}}$ = 1.07 × ${J}_0^{\textrm{KA}}$ − 0.28	0.91	${J}_{45}^{\textrm{RA}}$ = 1.46 × ${J}_{45}^{\textrm{KA}}$ − 0.03
827	18 − 59 (44 ± 12)	Current study	0.77	${J}_0^{\textrm{RA}}$ = 0.86 × ${J}_0^{\textrm{KA}}$ − 0.18	0.73	${J}_{45}^{\textrm{RA}}$ = 0.87 × ${J}_{45}^{\textrm{KA}}$ − 0.03

*RA* refractive astigmatism, *KA* keratometric astigmatism

Although there have been other studies which have proposed their own approach of Javal’s rule, some of their characteristics do not allow us to carry out a proper comparison. Javal’s results [4] are the first example. As they themselves explained, their results were derived from an empirical approximation, so we could not compare. Grosvenor et al. [6] accomplished their study using three different samples, either one or both eyes, and they did not consider oblique astigmatism. The disparities between the measurement conditions of their own samples, the divergence in criteria when choosing one or both eyes, and the elimination of certain types of astigmatism lead us to not be able to accurately simulate their conditions. Shankar et al. [9] conducted their study with preschool children under 5 years and divided it into two groups: astigmatism under or over 1.00 D. Low correlation coefficients were found for the group with low astigmatism and slope values, mostly for *J*_45_, significantly differed from the values usually reported in literature [4, 6–8]. Therefore, it was impossible to replicate it with our sample because of the age of the participants (our study does not include people under 7 years old). Likewise, Nagra et al. [14] assessed corneal astigmatism in a UK university population with Aladdin biometer. Nevertheless, the Aladdin biometer provides corneal values but does not have the capability to differentiate individual contributions from the anterior and posterior corneal interfaces. As a result, it was impractical to achieve an appropriate comparison.

**Fig. 3:**
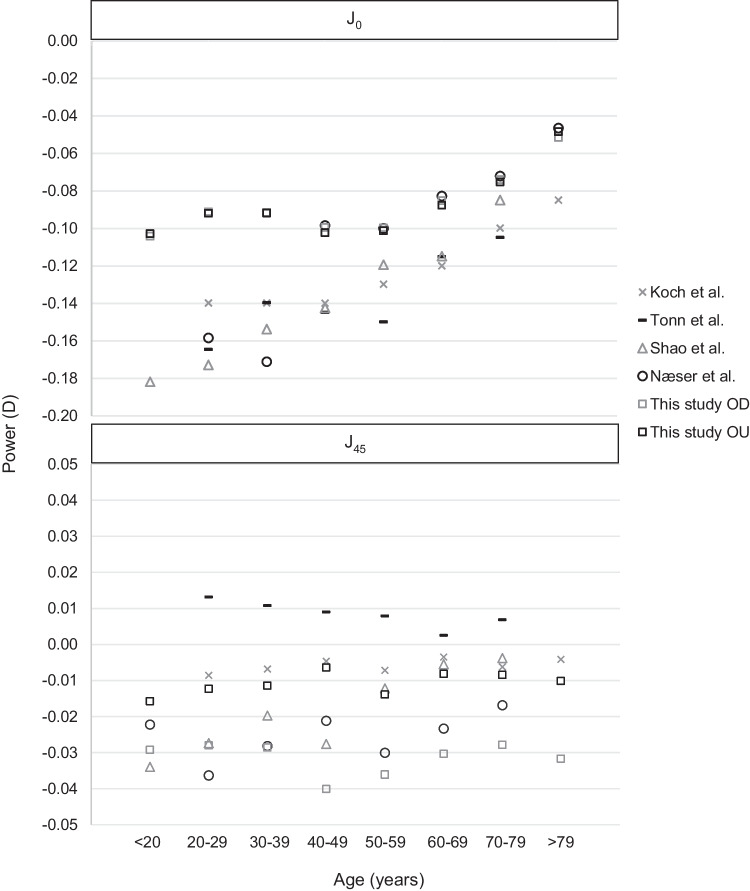
Posterior corneal astigmatism magnitude for *J*_0_ and *J*_45_ by age group for each study. OD, right eye; OU, both eyes

Some authors have evaluated the mean value of PCA based on the age of the subjects [19, 20, 22, 26]. The results obtained by these authors have been converted to a power vector notation for a more suitable comparison with the results from our study (Fig. 3). Koch et al. [19] and Tonn et al. [20] used both eyes in their samples, so both eyes in our sample are also included just at this point for comparison purposes with the above mentioned studies. As can be observed, differences for *J*_0_ component between results of the current study using both eyes and results from Koch et al. and Tonn et al. fluctuate between a minimum of 0.03 D in subjects within the age 70–79 years and a maximum of 0.07 D in the range of 20–29 years. These results imply a high level of agreement between our study and the previous ones. For the *J*_45_ component, the largest difference was observed for the 20–29 age, resulting in 0.02 D. Regarding the results obtained by Shao et al. [22] and Næser et al. [26] where only right eyes were evaluated, larger differences were observed in subjects under 39 years of age, with a maximum value of 0.08 D for *J*_0_ and a −0.02 D for *J*_45_. These discrepancies became smaller while the age of the subjects increases.

It is worthy to note that astigmatism should be analyzed only by using one eye due to, among others [34, 35] the possible presence of mirror symmetry between eyes in a same subject [24, 34]. This is the main reason why this study only considers both eyes to perform a similar comparison with preliminary studies but eliminate left eye measurements before any other calculation.

In summary, this study has developed new modifications of Javal’s rule in power vector formalism and introducing an additional, previously unexplored factor: the age of the subject. Furthermore, an evaluation of ACA and PCA has been conducted, finding a high correlation between both parameters which remains stable over the years. More complete Javal’s rules relating not only the KA but also the TCA with RA have been developed according to the subject’s age, overcoming the problems with oblique astigmatism. Developing a specific Javal’s rule depending on the age will help ophthalmologists and optometrists to obtain more accurate predictions of RA than employing previous models. Moreover, this study has conducted a detailed comparison to previous studies about Javal’s rule and PCA under the same conditions.

### Fundings

This study was funded by The Spanish Ministerio de Universidades (grant number FPU20/05624).
